# Supplementary material for: Serum neuron-specific enolase (NSE) is associated with the overall survival of colorectal cancer: a retrospective study
Source: PeerJ. 2024 Nov 22;12:e18617. doi: 10.7717/peerj.18617 (PMC11587878; doi:10.7717/peerj.18617)
Supplement: Supplemental Information 1 [file peerj-12-18617-s001.docx]

**Table S1:**

**Univariate Cox model fitting results**

| **Covariates** | **Univariate Cox Model** | |
| --- | --- | --- |
|  | **Crude HR (90% CI)** | ***p* value** |
| Sex (Male) | 1.09 (0.83-1.42) | 0.61 |
| Age at diagnosis (+5 years) | 1.06 (1.00-1.13) | 0.09 |
| Smoking history (Yes) | 0.88 (0.67-1.16) | 0.45 |
| Alcohol drinking history (Yes) | 0.88 (0.66-1.16) | 0.44 |
| BMI (+1 kg/m^2^) | 0.89 (0.85-0.94) | <0.01 |
| Chemotherapy (Yes) | 0.82 (0.63-1.06) | 0.21 |
| Curative operation (Yes) | 0.25 (0.18-0.34) | <0.01 |
| Clinical stage (Stage III-IV) | 6.73 (4.31-10.52) | <0.01 |
| Measure time of laboratory (+1 day) | 0.98 (0.84-1.14) | 0.82 |
| NLR (+1) | 1.24 (1.17-1.31) | <0.01 |
| ALB (>=35 U/L) | 0.12 (0.08-0.18) | <0.01 |
| AFP (>=8.78 ug/L) | 1.36 (0.43-4.39) | 0.66 |
| CEA (>=5 ug/L) | 4.19 (3.09-5.68) | <0.01 |
| CA125 (>=35 kU/L) | 4.04 (2.90-5.63) | <0.01 |
| CA19-9 (>=37 kU/L) | 2.94 (2.24-3.85) | <0.01 |
| NSE (>= 12.93 ng/mL) | 2.41 (1.84-3.17) | <0.01 |
