# Supplementary figures and images for: Serum neuron-specific enolase (NSE) is associated with the overall survival of colorectal cancer: a retrospective study
Source: PeerJ. 2024 Nov 22;12:e18617. doi: 10.7717/peerj.18617 (PMC11587878; doi:10.7717/peerj.18617)

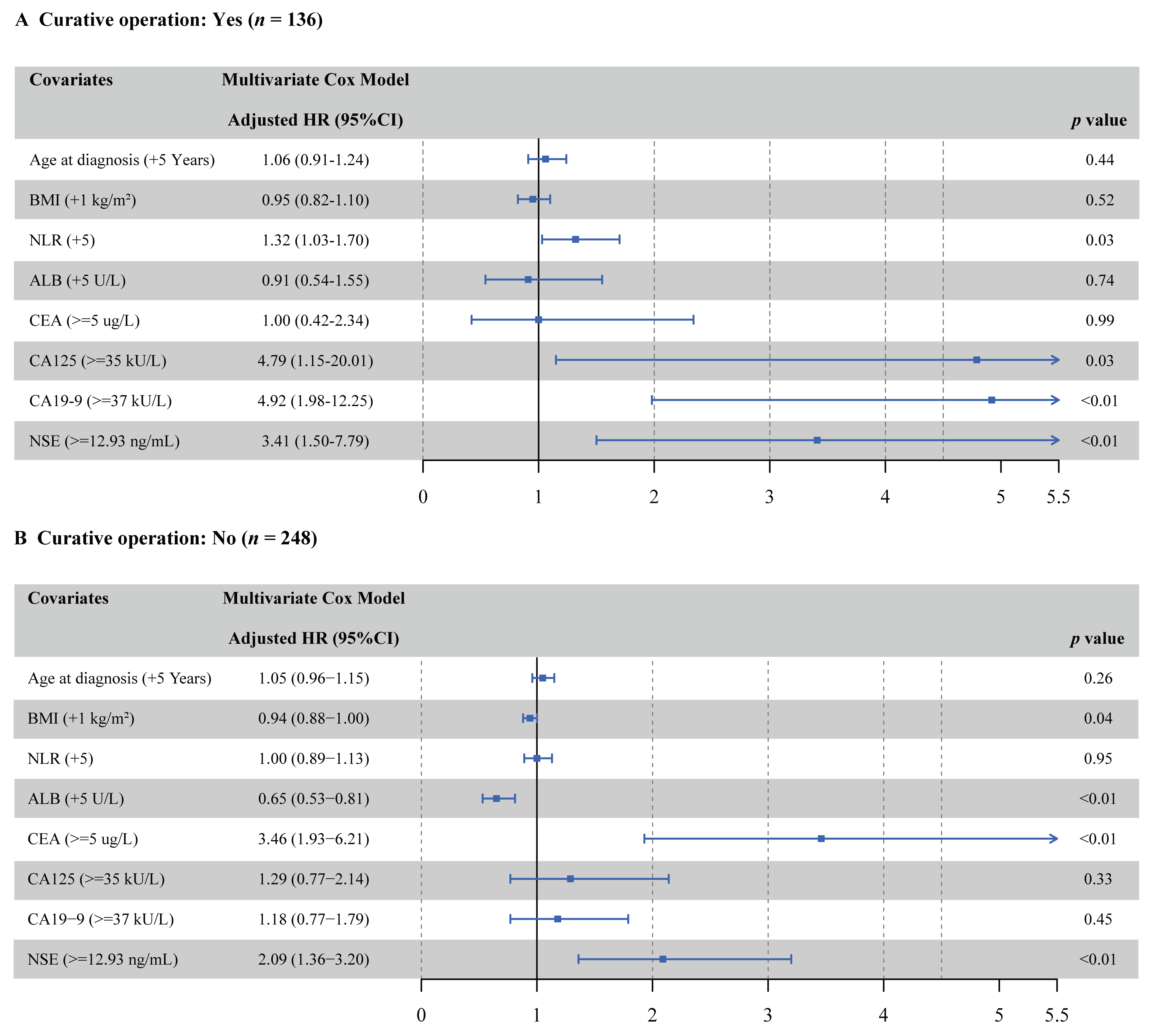

Supplement: Supplemental Information 2 [file peerj-12-18617-s002.png]
